# A Daily Cup of Tea or Coffee May Keep You Moving: Association between Tea and Coffee Consumption and Physical Activity

**DOI:** 10.3390/ijerph15091812

**Published:** 2018-08-22

**Authors:** Luciana Torquati, Geeske Peeters, Wendy J. Brown, Tina L. Skinner

**Affiliations:** 1 Centre for Research in Exercise, Physical Activity, and Health, School of Human Movement and Nutrition, The University of Queensland, Queensland 4072, Australia; wbrown@uq.edu.au (W.J.B.); t.skinner@uq.edu.au (T.L.S.); 2 Global Brain Health Institute, University of California, Oakland, CA 94158, USA; geeske.peeters@gbhi.org; 3 Trinity College Dublin, Dublin D02 PN40, Ireland

**Keywords:** caffeine, physical activity, fatigue, mediation analysis, middle-age

## Abstract

Physical activity (PA) is an independent predictor of mortality and frailty in middle-aged women, but fatigue remains a major barrier in this group. While caffeine intake has been associated with reduced exertion and perceived fatigue, it is not well understood whether consumption of naturally caffeinated drinks is associated with physical activity. The aim of this study was to determine whether habitual consumption of coffee and tea is associated with participation in physical activity. Women (*n* = 7580) from the Australian Longitudinal Study on Women’s Health were included in this investigation. Participants reported average tea and coffee intake over the last 12 months and usual PA. Logistic regression models were adjusted for relevant health and lifestyle confounders, and Sobel test was used for mediation analysis. Participants who consumed 1–2 cups of coffee/day were 17% more likely to meet the recommended 500 metabolic equivalent (MET).min/week than women who had <1 cup/day (odds ratio (OR) 1.17, 95% confidence interval (CI) 1.04–1.32). Participants who reported drinking either 1–2 cups or >3 cups/day of tea were 13–26% more likely to meet 500 MET.min/week than those who had <1 cup/day (OR 1.26, 95% CI 1.08–1.46 and OR 1.13, 95% CI 1.01–1.26, respectively). Tiredness and energy mediated associations between intake of coffee (fully) and tea (partially) and PA. Middle-aged women who drink 1–2 cups of coffee or >1 cup of tea/day are more likely to meet the moderate-to-vigorous PA guidelines than those who drink <1 cup/day. Future research is warranted to investigate causality and effects of specific coffee and tea amounts.

## 1. Introduction

Physical activity (PA) is an independent predictor of mortality and is inversely associated with physical decline in middle to late age [1,2]. Yet many factors influence physical activity engagement, with fatigue and tiredness being among the main barriers [3]. Caffeine, a naturally occurring alkaloid widely found in coffee and tea [4], has been shown to reduce perceived fatigue and tiredness [5]. Coffee consumption is associated with many health effects, including lower risk of type 2 diabetes, some cancers, dementia, and Alzheimer’s disease [6,7,8]. These health effects are believed to be explained not only by antioxidant compounds in coffee but also by its caffeine content. While caffeine properties could affect fatigue and tiredness, it is not well understood whether this could indirectly encourage PA participation or whether consumption of naturally caffeinated drinks is associated with physical activity. 

Caffeine is an adenosine A1 and A2A receptor antagonist leading to increased dopamine and noradrenaline release [9]. This property may be responsible for caffeine’s effects on feelings of wakefulness and alertness and decreased perceived exertion and pain during exercise [9].

Caffeine effects have also been associated with decreased exertion and increased enjoyment of exercise [10]. However, whether associations exist between caffeine intake and PA is yet unclear. In a randomized controlled trial (RCT), caffeine intake was associated with improved self-determined exercise duration after two weeks, but it had no effect on PA intensity [11]. Further, a meta-analysis of RCTs concluded that caffeine intake reduced perceived physical exertion during exercise, resulting in longer exercise time [12]. Likewise, caffeine ingestion before exercise resulted in participants having a more positive experience during prolonged cycling and reduced muscle pain after exercise [13]. These effects could potentially promote PA, as improved affective response to exercise has been associated with future engagement in PA [14]. Current evidence is limited on acute effects of caffeine supplementation, while little is known on whether usual intake of natural caffeine sources (e.g., tea and coffee) could promote PA participation or be associated with PA levels.

Given the limited evidence of caffeine’s effects on PA and its high availability in tea and coffee, the purpose of this study was to assess whether there is a relationship between caffeine consumption from tea and coffee (cups/day) and PA levels, and whether caffeine’s effects on tiredness and energy level mediate this relationship. We hypothesize that consumption of coffee and tea will be associated with higher PA participation.

## 2. Materials and Methods

Data presented in this study are from participants in the cohort born in 1946–1951 of the Australian Longitudinal Study on Women’s Health (ALSWH), a prospective study of the health and well-being of 3 generations of women [15]. Samples were randomly drawn from the national Medicare health insurance database, which included all Australian citizens and permanent residents, with intentional overrepresentation of women from rural and remote areas. Further details about the study are available at www.alswh.org.au. The study was approved by the ethics committees of the University of Newcastle and the University of Queensland, with all participants providing informed consent. 

In 1996, 13,715 women responded to the baseline survey. Of these, 9151 responded and completed the seventh follow-up survey in 2013 (80.9% of those eligible). Reasons for nonresponse included death (*n* = 650), withdrawal (*n* = 1652), frailty (*n* = 100), or not completing the survey (*n* = 2162). We analyzed data from the 2013 survey, as this is the most recent survey with questions on beverage consumption, PA participation, and physical health. Participants with missing values for coffee (*n* = 104) or tea (*n* = 144) consumption, level of PA (*n* = 312), level of energy (*n* = 84) or tiredness (*n* = 41), or any of the covariates (*n* = 576) were excluded from the analysis. Given the aim of this study, participants either with missing values or who responded to the question “Does your health limit you walking more than 500 m?” with “limited a lot” were excluded (*n* = 151). Therefore, data from 7580 participants were included in the analysis. 

### 2.1. Caffeinated Drinks Intake Assessment

The ALSWH study uses the Dietary Questionnaire for Epidemiological Studies (DQES) version 2. This food frequency questionnaire assesses usual consumption of 80 foods and beverages over the preceding 12 months using a 10-point frequency scale, and has been previously validated in middle-aged women [16]. Nutrient intakes were computed from Nutrient Tables (NUTTAB) 1995, a national government database of Australian foods [17], using software developed by the Cancer Council of Victoria. Spearman correlation coefficient for reproducibility ranged from 0.28 for vitamin A to 0.78 for carbohydrate when compared to weighted food records [16]. These values are comparable to those reported in similar larger studies, such as the European Prospective Investigation into Cancer and Nutrition (EPIC) study, which reported correlation coefficients between 0.5 and 0.7 [18]. 

Participants were asked to indicate their frequency of consumption of diet and regular cola drinks, carbonated and noncarbonated drinks, milk, juice, tea, coffee, and water in the last 12 months. As coffee and tea are the largest contributors of caffeine to the diet and in this age group [19], these beverages were selected for analysis as a proxy for caffeine intake. Frequency of intake of each beverage was categorized as <1 cup/day (with answers “never”, “<1 cup/month”, “1–3 cups/month”, “1 cup/week”, “2 cups/week”, “3–4 cups/week”); 1–2 cups/day (with answers “5–6 cups/week”, “1 cup/day”, “2 cups/day”); and ≥3 cups/day (“3 or more cups/day”).

### 2.2. Physical Activity

Participants were asked to report their frequency and duration of walking (e.g., for exercise or transport), and performing moderate leisure-time activities (e.g., swimming, recreational) and vigorous leisure-time activities (i.e., activities that make you breathe harder or puff and pant) in the previous week. Time spent in each activity (minutes/week) was multiplied by a metabolic equivalent (MET) score that reflected the average intensity of the activities in that category: 3.33 for walking and moderate leisure-time activities and 6.66 for vigorous leisure-time activities [20]. To estimate PA levels, MET.min/week from walking and moderate and vigorous leisure-time activities were summed. Consistent with the Active Australia protocols [21], outliers for this summary score (seen in 0.4% of women) were truncated at 40 h/week. Scores were categorized according to level of PA: inactive (0 MET.min/week), low (1–499 MET.min/week), moderate (500–1000 MET.min/week), and high (>1000 MET.min/week). The moderate and high groups were considered to be meeting the Australian Guidelines for Physical Activity [20].

### 2.3. Tiredness and Energy Levels

To assess feelings of tiredness and energy, participants were asked how much time during the past 4 weeks they either felt tired or had a lot of energy. These questions were taken from the SF-36 vitality subscale [22]. Responses were categorized as “most of the time” (with answers “all the time”, “most of the time”, “a good bit of the time”); “a little” (“some of the time”, “a little of the time”); and “never” (“none of the time”). We also created a dichotomous variable “most of the time” (with answer “all the time/most of the time/a good bit of the time”) and “little of the time” (“some of the time/little of the time/none of the time”), as discussed in the Statistical Analysis section.

### 2.4. Health Variables

Body mass index (BMI) (kg/m²) was calculated using self-reported body mass and height. Smoking was assessed by asking participants whether they currently smoke; answers were “never smoked”, “ex-smoker”, or “smoker.” The last category included all current smokers regardless of the number of cigarettes/day. Diabetes, hypertension, and cardiovascular disease were assessed by asking, “In the past 3 years, have you been diagnosed with or treated for…” Depressive symptoms were assessed using the 10-item Center for Epidemiological Studies – Depression scale (CES-D), with scores ranging from 0 to 30, higher scores indicating greater depressive symptoms [23]. 

### 2.5. Statistical Analysis

Descriptive statistics were used to summarize sample characteristics for the total group and by the women’s consumption of each beverage. Continuous variables are presented as mean and standard deviation (SD). Categorical variables are presented as percentages, and comparisons between levels of consumption each beverage were tested using the chi-squared test. The associations between coffee and tea intake (cups/day), and PA levels were analyzed using general linear models (GENLIN function). PA was the dependent dichotomous variable, with the outcome being meeting the PA guidelines. Potential confounders were selected based on previous studies, including smoking, age, BMI, education, occupation, cardiovascular disease, diabetes, and depressive symptoms, and assessed for interactions with the dependent and independent variables. The fully adjusted model included only those variables that resulted in >10% change in the regression coefficient of the crude model. Level of tiredness and feeling energized were not included in the model as confounders, when these were the outcomes of logistic regression. 

Levels of tiredness and feeling energized were considered potential mediators of the relationship between coffee/tea intake and meeting PA guidelines. Mediation analyses were conducted using separate logistic regression models, with cups of tea or coffee per day as independent variables (IV) and meeting PA guidelines as the dependent variable (DV). The models included the binary mediator variables “feeling tired” (yes/no; Model 1) and “feeling energetic” (yes/no; Model 2). The procedures described in Preacher and Hayes [24] and MacKinnon et al. [25] were used to assess changes in the regression coefficient between IV and DV with and without including the mediator in the model (M1 or M2). The Sobel test was used to assess whether the indirect effect of the mediator on the IV–DV relationship was significantly different from zero [24]. All analyses were performed using IBM^®^ SPSS^®^ Statistics Version 20.0 (Armonk, NY, United States). *p*-values were based on 2-sided tests and were considered statistically significant at *p* < 0.05.

## 3. Results

Participants’ (*n* = 7580) characteristics are displayed in Table 1. On average, participants were 65 years old, predominantly nonsmokers (93.3%), with a relatively equal distribution across the three BMI categories (39.5% normal, 35.0% overweight, 24.2% obese). Hypertension, diabetes, and cardiovascular disease were present in 33.4%, 7.7%, and 5.3% of participants, respectively. When asked about their energy levels and tiredness in the last four weeks, 62.5% reported feeling energized most of the time and 72.8% reported feeling tired a little of the time. A majority of the participants met the PA guidelines, and one in three reported drinking less than one cup of coffee or tea per day.

Table 2 shows the results of cross-tabulations for the association between coffee intake, PA level, and frequency of feeling tired or energetic. Statistically significant associations were found between coffee intake and MVPA and feeling energetic, and between tea intake and MVPA, tiredness, and feeling energetic. Participants who consumed 1–2 cups of coffee (64.8%) or tea (64.1%) per day were more likely to meet the MVPA guidelines than those who drank <1 cup of coffee (60.0%) or tea (58.1%) per day. Compared with participants who drank <1 cup a day, participants who reported >1 cup of coffee or 1–2 cups of tea per day were less likely to feel tired most of the time (18.3%, 17.5%, and 17.1%, respectively). The highest proportion of participants who reported feeling energetic most of the time (65.4% and 64.2%, respectively) was seen in those who reported 1–2 cups of coffee or tea per day.

### 3.1. Coffee or Tea Intake and Physical Activity

The relationship between consumption of coffee or tea and the odds of meeting PA guidelines are shown in Table 3. Participants who drank 1–2 cups/day of coffee were 23% more likely to meet the MVPA guidelines than women who drank <1 cup/day (odds ratio (OR) 1.23, 95% confidence interval (CI) 1.09–1.39). This relationship remained significant after adjusting for all possible confounders (OR 1.17, 95% CI 1.04–1.32). 

The results in Table 3 show that participants who consumed either 1–2 or >3 cups of tea per day were almost 20–30% more likely to meet the guidelines than those who drank <1 cup/day (OR 1.30, 95% CI 1.14–1.49 and OR 1.18, 95% CI 1.06–1.30, respectively). When adjusting for all possible confounders, the relationship remained significant for both categories, but the odds attenuated to 13–26% (OR 1.26 95% CI 1.08–1.46 for 1–2 cups/day, OR 1.13 95% CI 1.06–1.26 for >3 cups/day).

### 3.2. Mediation Analysis for the Relationship between Coffee and Tea Intake and PA

**Figure 1:**
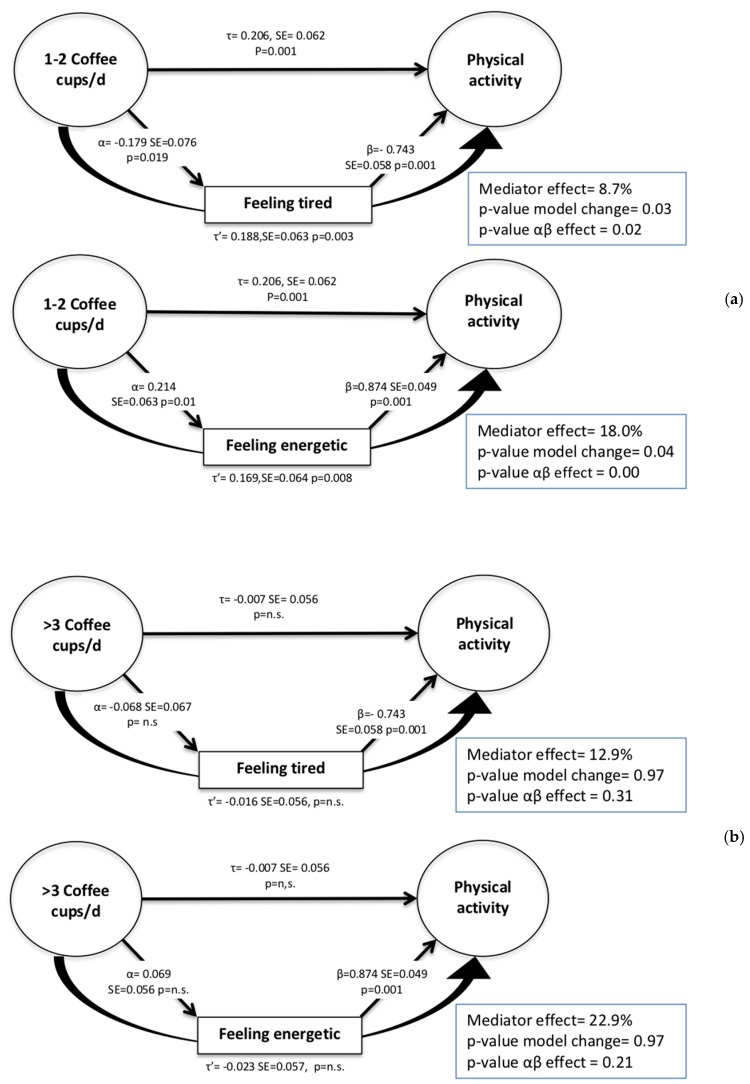
Mediation analysis for the relationship between coffee consumption and meeting physical activity guidelines for (**a**) 1–2 cups of coffee/day and (**b**) >3 cups of coffee/day, with correlation coefficients before (τ) and after (τ’) including the mediator in the model. Mediator effect: (τ − τ’)/τ = % difference in the regression coefficient for the independent variable (coffee intake) on the dependent variable (physical activity) between the models. *p*-value model change = tests the hypothesis that the direct effect of IV on DV is statistically significant after adjusting for the mediator; *p*-value αβ effect = tests the hypothesis that the indirect effect of the mediator on the IV–DV relationship is significantly different from zero.

Figure 1 shows the mediation effects of tiredness and energy in the association between coffee intake and PA level. “Feeling tired” explained 8.7% and 12.9% of the relationship between 1–2 cups and >3 cups of coffee per day and meeting PA guidelines, respectively. The Sobel test showed significant independent associations between “feeling tired” and both meeting PA guidelines and coffee intake. The change in the model after including the mediator was significant only for 1–2 cups of coffee per day. Thus, “feeling tired” was a mediator of the association between 1–2 cups of coffee per day and meeting PA guidelines. Likewise, “feeling energetic” was a significant mediator of the association between 1–2 cups of coffee per day and meeting PA guidelines. Both the model change (18%) and the independent effect of the mediator on coffee intake and meeting PA guidelines were statistically significant. On the other hand, these values were not significant for the category of >3 cups of coffee per day.

**Figure 2:**
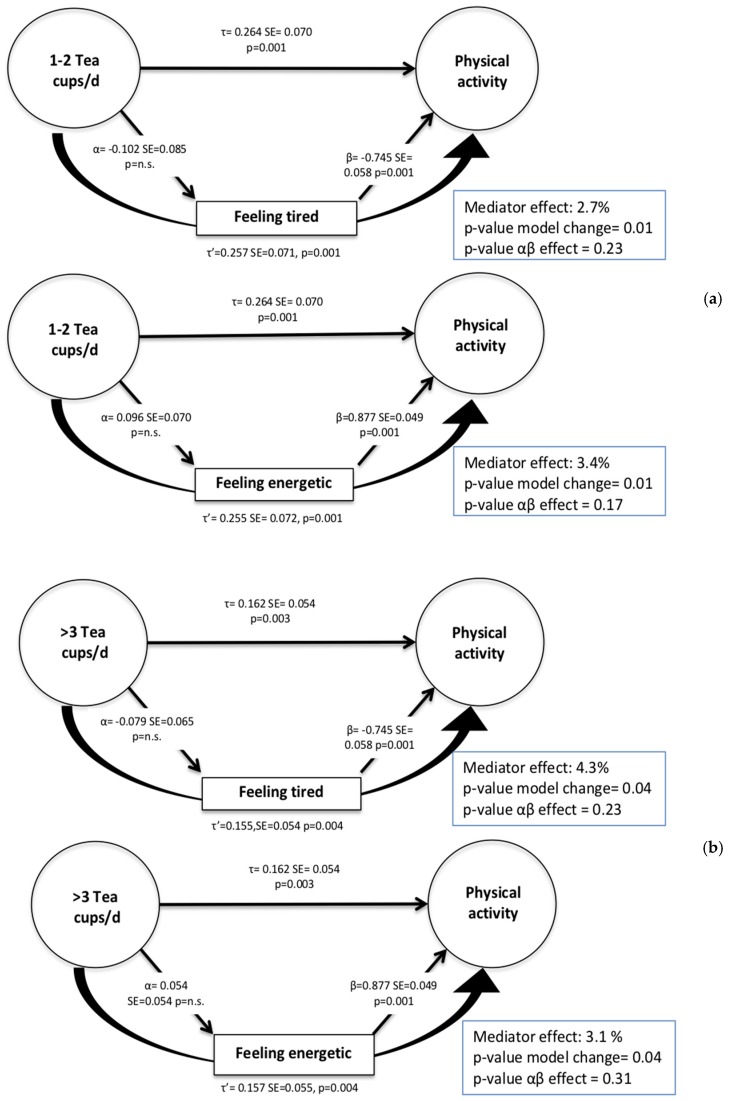
Mediation analysis for the relationship between coffee consumption and meeting physical activity guidelines (**a**) 1–2 cups of tea/day and (**b**) >3 cups of tea/day, with correlation coefficients before (τ) and after (τ’) including the mediator in the model. Mediator effect: (τ − τ’)/τ = % difference in the regression coefficient for the independent variable (tea intake) on the dependent variable (physical activity) between the models. *p*-value model change = tests the hypothesis that the direct effect of IV on DV is statistically significant after adjusting for the mediator; *p*-value αβ effect = tests the hypothesis that the indirect effect of the mediator on the IV–DV relationship is significantly different from zero.

Figure 2 describes the mediation effects of tiredness and energy in the association between tea intake and PA level. “Feeling tired” explained 2.7% and 3.4% of the relationship between 1–2 cups and >3 cups of tea per day and meeting PA guidelines. Conversely, “feeling energetic” showed significant mediation effects in the relationship between meeting PA guidelines and 1–2 cups (3.4%) and >3 cups (3.1%) of tea per day. However, the Sobel test for the independent effect of both potential mediators on the independent variable (IV) and dependent variable (DV) relationship was not significant for either category of tea intake. For these reasons, these variables had partial mediation effects in the relationship of IV–DV.

## 4. Discussion

In this cross-sectional study of Australian women, we found that 1–2 cups of coffee or tea per day was associated with 17–26% increased likelihood of meeting PA guidelines. Coffee and tea drinkers were less likely to feel tired and more likely to feel energetic. The relationship between coffee intake and PA seemed fully mediated by participants’ level of tiredness or energy, and such mediation was partial for tea intake. 

In the present study, the relationship between coffee/tea intake and PA level was not linear, as 1–2 cups but not >2 cups was associated with higher PA, suggesting a parabolic association. However, this nonlinear relationship could be due to participants drinking more coffee or tea because they felt tired or drinking little to no coffee/tea as they felt energetic and did not feel the need to drink these beverages. 

Therefore, the relationship between caffeine and PA might be mediated by energy level. Previous longitudinal and intervention studies indicated that increased PA levels lead to improved feelings of vitality and energy [26]. A study done with all three ALSWH cohorts showed that weekly PA volume was associated with increased levels of vitality in a dose-response relationship [27] and reduced feelings of fatigue [28]. On the other hand, studies show that fatigue and mood can undermine individuals’ intention to participate in PA, which is in line with our findings of feeling tired being associated with less PA [3,29]. Given the known stimulant effects of caffeine in the current literature [5] and the results of the present analysis, the relationship between caffeine intake and PA is likely to be mediated by reduced fatigue and increased feelings of vitality/energy. Yet, because of the cross-sectional nature of this study and the potential effects of other mediators not assessed here, it is possible this association might be bidirectional.

Promoting physical activity and exercise is important across all life stages, but it is particularly critical in middle age in order to maintain functionality and independence and reduce the risk of sarcopenia later in life [20]. The results of this study suggest that consumption of caffeine in coffee or tea could promote PA in middle-aged women by reducing some PA barriers, such as fatigue and lack of energy. Further, consumption of coffee and tea during middle age seems to have other health benefits, as it was shown to be a predictor of reduced risk for dementia and Alzheimer’s disease in late life [30]. Therefore, given the several proposed caffeine benefits from longitudinal studies, further investigation with intervention studies in this age group is warranted. Well-designed intervention studies should explore the potential effect of encouraging tea and coffee consumption when people are feeling tired or have low energy in order to increase MVPA, while controlling for confounding factors such as usual PA level, baseline cognitive and physical function, and concurrent caffeine intake from other food sources.

This study has several strengths, including the use of ALSWH data, which includes a large representative sample of Australian women, and adjusting for a broad range of confounding variables. This large and random sample allows for generalizability of results of women in this age group, as a sub-analysis including all participants with missing values showed similar results to those in the final sample. 

The main limitation is the reliance on self-reported data, which is subject to recall and measurement bias. The strength of the associations may have been underestimated as a result, with possible misclassification. However, the PA measures used in this cohort have been reported to have adequate reliability and validity [21]. Another limitation is the use of cups of coffee and tea as a proxy for caffeine intake, leading to estimations rather than actual measures of caffeine intake. Variations in the size of a cup of tea or coffee, brand, quality, preparation, and caffeine content of these beverages may also have impacted the variability of actual caffeine intake within categories. Indeed, tea and coffee often vary quite markedly in regard to their caffeine content, with Desbrow et al. [31] reporting a range of 25–214 mg of caffeine in espresso sold by a sample of commercial coffee vendors. The cross-sectional associations shown here point to complex relationships between caffeine intake and PA, but given the nature of this study, we cannot conclude whether caffeine intake is a determinant of PA or the reverse is true. Including only women in this study might limit the generalization to men, although we anticipate similar associations in both sexes. Further, other confounders influencing both coffee/tea intake and physical activity, such as social isolation or medical conditions, were not assessed; therefore, residual confounding cannot be ruled out.

Insight into the direction of these relationships is important, as it may reveal whether increasing tea or coffee intake can improve PA levels of middle-aged women. Alternative but complementary methods to the ones presented here, such as objective measurements or diet and activity diaries, could provide more insightful data in long-term cohort studies. Understanding the direction of this complex relationship may assist in providing more accurate recommendations for PA promotion strategies. 

## 5. Conclusions

Drinking 1–2 cups of coffee or >1 cup of tea per day was associated with 17% and 13–26% increased likelihood of meeting PA guidelines, respectively, in middle-aged women. This association was partly mediated by reduced fatigue and increased energy levels. Consumption of caffeine in coffee or tea could be a way to modify tiredness and low energy levels as a barrier to MVPA in middle-aged women. Randomized controlled trials would be required to explore this hypothesis. These could explore a potential dose-response relationship by controlling the actual amount of caffeine intake through tea and coffee consumption.

## Figures and Tables

**Table 1:** Sociodemographic characteristics of 7580 women aged 64.7 ± 1.5 years included in the analysis. BMI, body mass index; PA, physical activity; MET, metabolic equivalent.

Characteristic	Categories	*n* (%)
BMI	Underweight	94 (1.2)
	Normal	2995 (39.5)
	Overweight	2655 (35.0)
	Obese	1836 (24.2)
Education level	No formal qualification	958 (12.6)
	School certificate	2397 (31.6)
	Higher school certificate	1263 (16.7)
	Trade/apprenticeship	258 (3.4)
	Certificate/diploma	1388 (18.3)
	University degree or higher	1316 (16.4)
Smoking	Never	4799 (63.3)
	Ex-smoker	2277 (30.0)
	Smoker	504 (4.7)
A lot of energy in the past four weeks	Never	380 (5.0)
	A little	2465 (32.5)
	Most of the time	4735 (62.5)
Tiredness in the past four weeks	Never	556 (7.3)
	A little	5515 (72.8)
	Most of the Time	1509 (19.9)
Hypertension	Yes	2533 (33.4)
	No	5047 (66.6)
Diabetes	Yes	583 (7.7)
	No	6997 (92.3)
Cardiovascular disease	Yes	405 (5.3)
	No	7175 (94.7)
Coffee intake (cups/day)	<1 cup	2360 (31.1)
	1–2 cups	2064 (27.2)
	>3 cups	3156 (41.6)
Tea intake (cups/day)	<1 cup	2306 (30.4)
	1–2 cups	1389 (18.3)
	>3 cups	3885 (51.3)
Meeting PA guidelines	(>500 MET.min/week)	4642 (61.2)

**Table 2:** Percentage of participants in each category of coffee and tea consumption and level of moderate-to-vigorous physical activity (MVPA), tiredness, and energy (*n* = 7580).

Variables	Category	Coffee (Cups/Day)			Tea (Cups/Day)		
		<1	1–2	≥3	<1	1–2	≥3
**Level of MVPA** Coffee χ^2^ = 21.39, *p* < 0.01 Tea χ^2^ = 21.28, *p* < 0.01	None	15.2	12.1	14.7	16.2	11.6	13.8
	Low	24.8	23.1	25.5	25.8	24.0	24.2
	Moderate	22.4	22.0	21.8	20.9	22.8	22.5
	High	37.6	42.8	38.0	37.3	41.5	39.5
**Meeting MVPA guidelines** Coffee χ^2^ = 15.38, *p* < 0.01 Tea χ^2^ = 16.11, *p* < 0.01	Yes	60.0	64.8	59.8	58.1	64.4	62.0
**Tiredness** Coffee χ^2^ = 5.60, *p* = 0.05 Tea χ^2^ = 10.85, *p* = 0.03	Most of the time	21.1	18.3	17.5	21.0	17.1	21.2
**Feeling energetic** Coffee χ^2^ = 13.84, *p* < 0.01 Tea χ^2^ = 3.29, *p* = 0.51	Most of the time	60.4	65.4	64.7	61.2	64.2	61.7

**Table 3:** Odds ratio for coffee or tea consumption and meeting physical activity guidelines (*n* = 7850).

Cups/day (*Reference*: <*1 Cup/d*)	Coffee			Tea		
	Crude	Model 1	Model 2 ^a^	Crude	Model 1	Model 2 ^b^
1–2 cups	1.23 (1.09–1.39)	1.17 (1.04–1.32)	1.14 (1.00–1.30)	1.30 (1.14–1.49)	1.26 (1.08–1.46)	1.23 (1.05–1.40)
≥3 cups	0.99 (0.89–1.10)	1.03 (0.92–1.16)	0.98 (0.87–1.10)	1.18 (1.06–1.30)	1.13 (1.01–1.26)	1.09 (0.98–1.22)

Model 1: Adjusted for age, education, occupation, smoking, BMI, cardiovascular disease, diabetes, depression, tiredness, and feeling energetic; Model 2: Adjusted only for variables changing >10% regression coefficient of the crude model. ^a^ Education, BMI, tiredness, feeling energetic. ^b^ Education, smoking, BMI.
